# Which Biomarker(s) Augment the Diagnostic Value of the Positive Exercise Electrocardiography Test: Systemic Inflammatory Index, Plasma Atherogenic Index, or Monocyte/HDL-C Ratio?

**DOI:** 10.3390/jcm12206440

**Published:** 2023-10-10

**Authors:** Gokhan Ergun, Selami Demirelli

**Affiliations:** Department of Cardiology, Kayseri City Training and Research Hospital, 38080 Kayseri, Turkey; demirelli23@yahoo.com

**Keywords:** chronic coronary syndromes, exercise electrocardiography test, monocyte/HDL-C ratio, positive predictive value

## Abstract

The exercise electrocardiography test (EET) is still used before coronary angiography in the diagnosis of chronic coronary syndromes. This study aimed to demonstrate the value of the combination of a positive EET with the systemic inflammatory index (SII), the plasma atherogenic index (PAI), and the monocyte/HDL-C ratio (MHR) in the determination of obstructive coronary artery disease (CAD). This single-center, retrospective study included 540 patients who underwent coronary angiography after ETT. The patients were separated into Group 1, comprising 434 patients with normal coronary arteries and non-obstructive CAD, and Group 2, including 106 with obstructive CAD. In Group 2, the patients were separated into SYNTAX ≤ 22 or ≥23. Glucose, low-density lipoprotein, white blood cells, and MHR were determined to be significantly higher in Group 2 (*p* < 0.05). According to the multivariate logistic regression analysis, age, gender, diabetes mellitus, and low-density lipoprotein were determined to be independent predictors of CAD. In the ROC curve analysis, a cut-off value of 12 for the MHR in the determination of obstructive CAD had a sensitivity of 60.4% and a specificity of 53.0%. The main result of this study was that a high MHR is an indicator of obstructive CAD in patients with positive EET and suspected CAD.

## 1. Introduction

As cardiovascular diseases are the leading cause of mortality worldwide [1], diagnostic strategies for these diseases are important. Although many diagnostic methods can be applied before invasive tests, especially in chronic cardiac syndromes, each has different advantages and disadvantages. In the European Society of Cardiology (ESC) guidelines published in 2020, it is recommended that after risk classification of chronic coronary syndromes, for patients with suspected coronary artery disease (CAD), tests such as stress echocardiography, computed tomography angiography (CCTA), stress cardiac magnetic resonance imaging, single photon emission computed tomography (SPECT), or positron emission tomography (PET) be performed before coronary angiography with class 1 indication, but the exercise electrocardiography test (EET) is recommended with class 2b indication when the above-mentioned tests cannot be performed [2]. EET has lost its former popularity and is no longer routinely used to investigate CAD. In a meta-analysis, it was reported that EET has 68% sensitivity and 77% specificity in the determination of obstructive CAD [3]. It was also shown in a study by Roiffman et al. that EET has equal value to that of other tests in the determination of obstructive CAD [4]. For many different reasons, such as the cost of other tests, local restrictions on other tests, and the fact that other tests are time-consuming, EET is performed before coronary angiography in the routine practice of many cardiology polyclinics. In recent years, several biomarkers have been associated with CAD, and there have been many studies of these. These biomarkers include the systemic inflammatory index (SII), plasma atherogenic index (PAI), and the monocyte/high-density lipoprotein (HDL-C) ratio (MHR).

SII is a marker of inflammation derived from platelet, neutrophil, and lymphocyte counts and was first described in hepatocellular carcinoma by Hu et al. [5]. It then became a biomarker used in studies in several areas. A previous study in the field of cardiovascular diseases determined that the SII was a better indicator for major cardiovascular events than traditional CAD risk factors in patients with coronary intervention [6]. There are also studies showing an association between elevated SII and the presence and severity of CAD. For example, two different studies have shown that SII is a good predictor of atherosclerotic burden [7,8]. In addition, Dziedzic et al. reported that SII was found to be higher in patients with 3-vessel disease [9]. All these data suggest that SII is closely associated with CAD.

The association of high triglyceride levels and low HDL levels with CAD has been previously demonstrated [10,11]. Therefore, PAI formed by the combination of these two markers has been the focus of interest in studies. PAI is a good indicator of atherosclerosis and cardiovascular disease risk [12,13]. Another study showed that the PAI could be a marker for the early diagnosis of cardiovascular disease in developing countries [14]. In two different meta-analyses including 10 and 14 studies, the association of high PAI values with CAD was revealed [15,16]. In conclusion, all these data demonstrated the association between PAI and CAD.

The MHR is another new biomarker that is often used in research and shows the balance between inflammatory stress and oxidative stress [17]. Significant results were found in studies of MHR with CAD. In a published review, MHR was found to be a predictor of atherosclerotic development and progression, as well as a marker of systemic inflammation [18]. MHR has also been shown to be associated with atherosclerosis burden and CAD severity [19,20]. In addition, its prognostic importance has been shown in different studies [21,22].

Taking the relationship between the SII, PAI, and MHR parameters and cardiovascular diseases and atherosclerosis into consideration, this study hypothesized that when EET was used together with these parameters, it could be helpful in the determination of obstructive coronary artery disease. If a positive relationship is determined between these parameters and obstructive coronary artery disease in patients with positive EET (pEET), better patient selection could be applied for the tests recommended with a class 1 indication before invasive intervention by ESC or could facilitate the selection of patients who are to undergo coronary angiography according to the EET result when these tests cannot be performed. This study aimed to determine the value of the combination of EET with three biomarkers in the determination of CAD.

## 2. Materials and Methods

**Figure 1:**
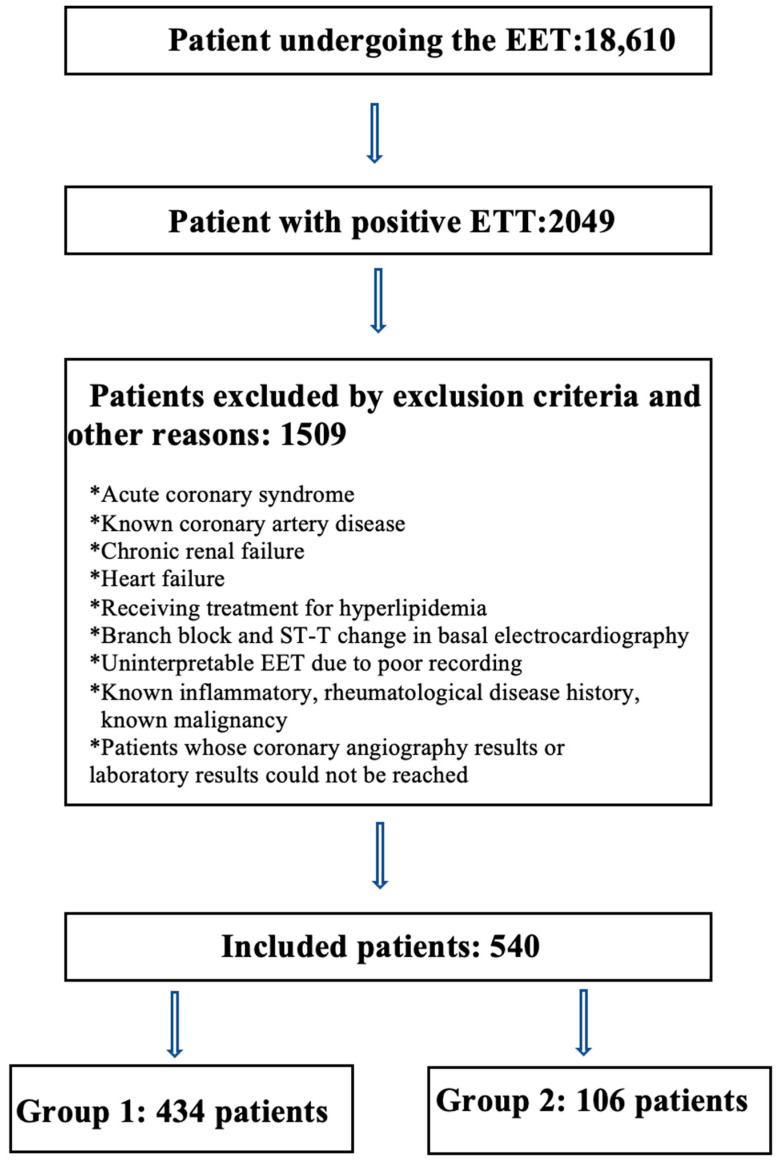
Study flow chart. ETT: exercise electrocardiography test, Group 1: normal coronary arteries and non-obstructive coronary artery disease, Group 2: obstructive coronary artery disease.

The patients for this study were selected from the 18,610 patients who underwent EET upon presentation at the Cardiology Polyclinic of Kayseri City Hospital between 2018 and 2021. A total of 2049 patients were identified with positive EET who underwent coronary angiography. Patients with an insufficient EET record, or biochemistry, hemogram, or angiography records not available from the patient files, were excluded from the study. Patients were also excluded if they had received treatment because of hyperlipidemia or had previously been diagnosed with CAD. Following the implementation of the exclusion criteria, a total of 540 patients were included in the study (Figure 1). Approval for the study was granted by the Ethics Committee of Kayseri City Hospital (decision no: 690, dated: 18 August 2022).

All the patients underwent EET with a GE device, according to the modified Bruce protocol. Before starting the test, resting heart rate, blood pressure, and 12-derivation electrocardiography were recorded in a supine and vertical position. At every minute during the test, heart rate, blood pressure, and electrocardiography were recorded. The electrocardiography records were evaluated according to the ACC/AHA guidelines [23]. In the exercise electrocardiography evaluation, patients who completed the test with >1 mm ST depression after 60–80 msn from the J point, and patients with typical angina or angina-equivalent symptoms, were accepted as EET-positive. The EET results of all the patients were evaluated by 2 experienced cardiology specialists.

In all the patients, selective coronary angiography was performed in multiple projections with the Judkins technique using a 6 or 7 French catheter with a right or left femoral approach. Iopromide (Ultravist-370^®^) or Iohexol (Omnipaque^®^ 350 mg/mL) was used as the opaque agent. The coronary angiography records were obtained using a GE device. The 540 patients included in the study were separated into 2 groups according to the coronary angiography results. Group 1 included 434 patients with normal coronary arteries and <50% narrowing in the epicardial arteries (non-obstructive coronary artery disease), and Group 2 included 106 patients with >50% narrowing in the epicardial arteries (obstructive coronary artery disease). In the Group 2 patients with vessel diameter > 1.5 mm, the SYNTAX score was calculated using the “syntaxscore.org” website, and the patients were separated into 2 groups, i.e., those with a SYNTAX score ≤ 22 or ≥23. All the coronary angiography results were evaluated by 2 experienced cardiology specialists.

Blood samples for the laboratory tests were taken from all the patients from an antecubital vein at 08:00–10:00 after 12 h of fasting and before the angiography. Evaluations were made of a comprehensive metabolic panel and full blood count (white blood cells, neutrophil, lymphocyte, monocyte, platelet, hemoglobulin, glucose, low-density lipoprotein, high-density lipoprotein, triglycerides, glomerular filtration rate). From these results, the SII was calculated as the platelet count multiplied by the neutrophil count and then divided by the lymphocyte count. The PAI was calculated with the logarithm of the triglyceride value divided by the HDL-C value (Log [triglyceride/HDL-C]) and MHR as the monocyte count divided by the HDL-C value.

The patients included in the study were those who were aged > 18 years who presented at the Cardiology Polyclinic with chest pain or equivalent symptoms, for whom EET was requested with an initial diagnosis of chronic coronary syndromes, and who underwent coronary angiography within 6 months of the test. Patients were excluded from the study if they had acute coronary syndrome, known CAD, chronic renal failure, heart failure, basal electrocardiography branch block and ST-T change, a history of inflammatory or rheumatological disease, or a known malignancy. Patients receiving treatment for hyperlipidemia were also excluded, as it could affect the PAI and MHR results.

### Statistical Analysis

Data obtained in the study were analyzed statistically using IBM SPSS for Windows version 23.0 software. Continuous variables were summarized as mean ± standard deviation (SD) or median (25th–75th percentage) values, and categorical values as number (n) and percentage (%). The conformity of numerical variables to normal distribution was assessed with the Shapiro–Wilk test. Differences between two groups of numerical variables were examined with the Student’s t-test when parametric test conditions were met and with the Mann–Whitney U-test when distribution was not normal. Comparisons of more than two groups of numerical variables were made with One-Way Analysis of Variance when parametric conditions were met and with the Kruskal–Wallis test when they were not met. The Chi-squared test was used to examine relationships between categorical variables. A value of *p* < 0.05 was accepted as statistically significant.

## 3. Results

Only 106 of 540 patients with pEET had obstructive CAD. Therefore, the positive predictive value of positive EET in detecting obstructive CAD was 19%.

The demographic data of all the patients in Group 1 and Group 2 are summarized in Table 1. No significant difference was determined between the two groups in respect to body mass index, systolic and diastolic blood pressure, pulse, and hypertension. Older age and male gender were determined at significantly higher rates in Group 2 than in Group 1 (*p* < 0.001, *p* < 0.001). Diabetes mellitus was observed more often in Group 2 than in Group 1 (*p* = 0.003). The laboratory parameters of the patients are also summarized also in Table 1. No statistically significant difference was determined between the two groups in respect to the triglyceride, neutrophil, lymphocyte, monocyte, hemoglobin, platelet, SII, and PAI values, and a difference was determined in respect to low-density lipoprotein, white blood cells, MHR, glomerular filtration rate, and HDL-C values. The glucose, low-density lipoprotein, white blood cells, and MHR values were determined to be significantly higher (*p* = 0.006, *p* = 0.033, *p* = 0.043, *p* = 0.019, respectively) and the glomerular filtration rate and HDL-C values were significantly lower (*p* = 0.001, *p* = 0.010, respectively) in Group 2 than in Group 1. In addition, although not statistically significant, the PAI value was found to be higher in Group 2 compared to Group 1. (0.21 ± 0.269 vs. 0.259 ± 0.274, *p* = 0.094).

**Figure 2:**
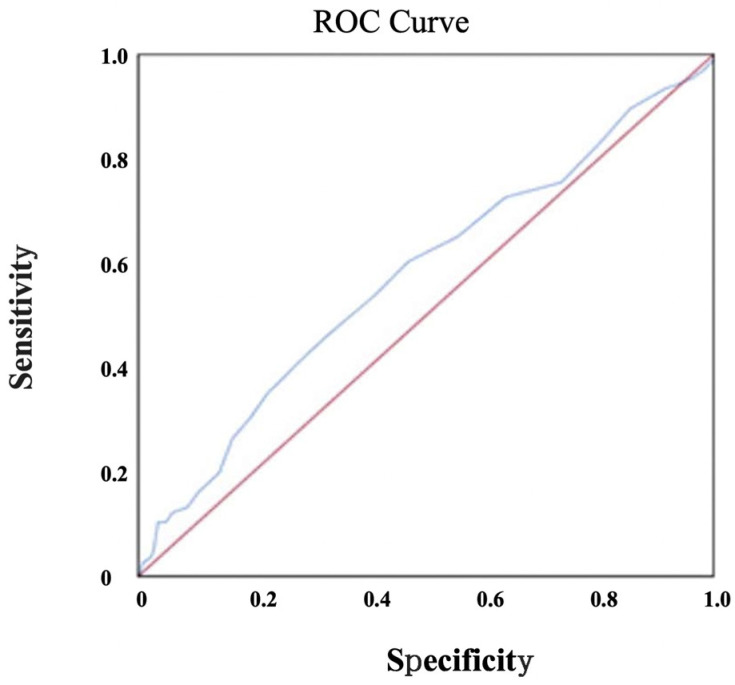
ROC analysis for MHR.

According to the results of the multivariate logistic regression analysis, age, gender, diabetes mellitus, and low-density lipoprotein were determined to be independent predictors for obstructive CAD (OR: 1.058, 95% CI: 1.034–1.084, *p* < 0.001; OR: 3.652, 95% CI: 2.137–6.239, *p* < 0.001; OR: 2.239, 95% CI: 1.285–3.903, *p* = 0.004; OR: 1.009, 95% CI: 1.002–1.015, *p* = 0.007, respectively) (Table 2). The ROC analysis results showed that a cut-off value of 12 for the MHR in the determination of obstructive CAD had 60.4% sensitivity and 53.0% specificity (Figure 2). The negative predictive value for MHR was determined to be 84.6%, and the positive predictive value was 23.9%.

The comparisons of the PAI, SII, and MHR values of the patients with obstructive CAD separated into two subgroups according to the SYNTAX score are shown in Table 3. In the SYNTAX ≥ 23 group, the PAI and MHR values were determined to be high (*p* = 0.543, *p* = 0.616, respectively) and the SII value was low, but the differences were not statistically significant (*p* = 0.711).

According to the results of coronary angiography, 13% of the patients were not revascularized and were given only optimal medical treatment, 54% underwent a percutaneous coronary intervention, and 33% underwent coronary arterial by-pass graft operation. As a result, percutaneous coronary intervention is the most common procedure in chronic coronary syndromes (Table 4). In addition, the most frequently obstructed vessels in the obstructive CAD group were the left anterior descending artery, circumflex artery, right coronary artery, intermediate artery, and left main coronary artery, and their percentages were 37%, 31%, 26%, 5%, and 1%, respectively (Table 4).

## 4. Discussion

The main result of this study was that a high MHR is an indicator of obstructive CAD in patients with pEET and suspected CAD. It was also determined that the positive predictive value of positive EET in the determination of obstructive CAD was 19%, and this value increased to 23.9% in patients with a high MHR.

In recent years, several biomarkers have been found to be associated with CAD, the most prominent of which are SII, PAI, and MHR. As a result of clinical and experimental studies, inflammation has been shown to have a critical role in the development and progression of atherosclerosis and CAD [24]. There are several mediators and cholesterol particles, primarily monocytes, in inflammation-causing atherosclerosis. During the development of atherosclerotic plaque, monocytes expressed from damaged endothelium are activated by being bound to adhesion molecules [25]. Then, the activated monocytes migrate to the arterial intima, where they become macrophages and, with lipid loading by phagocytosis of modified lipoproteins, form foam cells, which are characteristic of atherosclerosis [26]. In a review that included nine studies covering acute coronary syndrome and stable CAD, it was shown that monocytes play a key role in various stages from the onset of atherosclerosis to progression and in many areas of myocardial recovery and re-modeling after acute coronary syndrome [27]. When all these data are evaluated, the importance of monocytes in the development of atherosclerosis can be understood. 

Atherosclerosis is a dynamic process which is determined according to the result of inflammatory and anti-inflammatory balance. Just as monocytes, macrophages, mediators, and cholesterol trigger inflammation and atherosclerosis, some other cells and molecules with anti-inflammatory effects also slow down atherosclerosis. HDL-C is prominent as a molecule with a well-known anti-inflammatory effect. Previous studies have shown that HDL-C has a preventative effect against the atherosclerotic process [28,29] and that HDL-C reduces cardiovascular risk [30]. An analysis that included 68 studies determined a close relationship between HDL-C and non-HDL-C levels (in opposite directions) and CAD risk [31]. The anti-inflammatory effects and effects against atherosclerosis of HDL-C are explained by the decrease in adhesion and monocyte activation and regulation of endothelial adhesion molecule expression, reversing the effects of oxidized low-density lipoprotein and causing vasodilation with nitric oxide expression [32,33].

In light of these data, low HDL-C and high monocyte levels can be said to be indirect markers of inflammation and atherosclerosis. Therefore, it is quite natural that an elevated MHR, which is the inflammation/anti-inflammation ratio, provides information about atherosclerosis. There are several studies in the literature related to this. In one previous study, it was shown that a high MHR value was a risk factor for atherosclerosis and could be evaluated as a predictor of atherosclerosis development [34]. According to the National Health and Nutrition Examination Survey (NHANES) data, a positive correlation and clear relationship were determined between MHR and CAD in a study of 25,862 American adults between 2009 and 2018 [35]. Together with studies showing the presence of atherosclerosis, there are also studies related to disease severity. A previous study reported a relationship between high MHR values and a high SYNTAX score in stable CAD patients [19,36]. In the current study, a high MHR value in patients with pEET was determined to be an indicator of atherosclerosis and obstructive CAD. Unlike other studies, although the data obtained showed a positive correlation between MHR and CAD severity, a level of statistical significance was not reached. From the data obtained in this study, it was concluded that MHR is a good marker for obstructive CAD in patients with pEET, but this is not valid for CAD severity.

In studies that have used the SII calculated from these markers, a significant relationship has been determined between the SII and CAD severity in stable CAD [8,37]. In a study by Erdoğan M. et al. using the fraction flow reserve, the SII in patients with chronic coronary syndromes was determined to be an independent marker of hemodynamically significant coronary artery obstruction [38]. However, the data of the current study do not support the results of those studies. The current study results showed that the SII was not determined to be significant in respect to obstructive CAD and CAD severity in patients with pEET. In contrast to the previous studies, although not at a statistically significant level, the SII was determined to be low in the obstructive CAD group and the group with a high SYNTAX score. Therefore, it can be considered necessary to investigate the relationship between the SII and CAD, and there is a need for further, larger, comprehensive studies that will show the relationship between these.

In a meta-analysis of PAI, which is another marker, a relationship was found between high PAI values and CAD [15]. Wang et al. determined that high PAI was an independent risk factor for CAD and a high SYNTAX score [39]. According to the data obtained in the current study, although the PAI was determined to be high in the obstructive CAD group and the group with SYNTAX ≥ 23, these values were not statistically significant.

As mentioned before, ESC recommends ETT as an alternative to class 1 tests [2]. The main reason for this is that the diagnostic value of ETT in the detection of obstructive CAD is low compared to other tests. In a previous study, the sensitivity, specificity, positive predictive value (PPV), and negative predictive value (NPV) of pETT in detecting obstructive CAD were 71.4%, 90.4%, 13.5%, and 99.3%, respectively [40]. However, all other proposed tests have various limitations, advantages, and disadvantages and all have different sensitivity, specificity, PPD, and NPD values for detecting obstructive CAD. CCTA has been increasingly used in recent years and is an important test that provides anatomical information by non-invasively visualizing the coronary arteries. On the other hand, technological advances have made it possible to combine CCTA with fractional flow reserve (FFR) without any invasive intervention [41]. This new test is called CT-FFR and has provided the link between anatomical and functional non-invasive testing by demonstrating the hemodynamic significance of coronary lesions. The sensitivity, specificity, PPD, and NPD values of CCTA and CT-FFR for detecting obstructive CAD were 95%, 83%, 64%, and 99% [42], and 93.6%, 88.1%, 85.3%, and 94.9% [43], respectively.

Functional tests also play an important role in the diagnosis of obstructive CAD. Among these, myocardial perfusion scintigraphy (MPS) is frequently used because it shows ischemia and anatomically determines its location. Especially in patients who are unable to exercise and have ST–T abnormalities (left bundle branch block, paced rhythm, etc.) in the basal electrocardiogram (ECG), MPS is used quite frequently. MPS is a test that can be performed by exercise or pharmacological methods and shows the distribution of blood flow in the myocardium during rest and stress. MPS has two main modalities, known as MPS-SPECT and MPS-PET. While the sensitivity, specificity, PPD, and NPD values of MPS-SPECT are 66.5%, 82.6%, 71.4%, and 79.1% [44], these values are 90%, 90%, 96%, and 76%, respectively, in MPS-PET [45].

Stress echocardiography is another functional test that can be performed with physiological or pharmacological stress, and is also recommended by ESC with a class 1 indication. In a multicenter study, the sensitivity, specificity, PPD, and NPD values for stress echocardiography were 95.4%, 96.0%, 82.8%, and 99.0%, respectively [46].

As can be seen from all these data, the sensitivity values of ETT are lower than most of the other tests. In addition, another remarkable diagnostic value is the PPD value, which is significantly lower in ETT compared to other tests. The positive predictive value is an indicator of how many actual patients there are from those determined with a positive test result and is calculated with the formula of “number of correct positive tests/number of false and correct positive tests” [47]. Previous studies have determined different positive predictive values for pEET. In a previous review of seven studies, a mean positive predictive value of 21% was reported, varying from 5% to 46% [48], and in another study of athletes, it was reported to be 9% [49]. To increase the positive predictive value of EET, which has an extremely limited positive predictive value, age, ST depression type and amount, the number of derivations and affected derivations with ST depression, and how early ST depression occurred are used. In a study, it was emphasized that the positive predictive value of EET increased when the age was over 65 years, the amount of ST depression used was 2 mm, and the ischemic ST-segment recovery time was more than 3 min [50]. In the current study, the positive predictive value of pEET in the determination of obstructive CAD was determined to be 19%, and when evaluated together with MHR, this rate rose to 23.9%.

In our results, although PPD increased with the addition of MHR to ETT in the detection of obstructive CAD, it is still significantly lower than in other tests. For this reason, we think that the combination of ETT and MHR should not be used instead of other tests, but should be in patients with suspected obstructive CAD, as an aid in the selection of non-invasive tests to be requested, or in cases where other tests are not technically accessible.

There were some limitations to be considered in this study, primarily that it was retrospective in design, it was conducted in a single center, and the sample size was relatively small. Secondly, parameters associated with CAD, such as C-reactive protein, NT-proBNP, and (hs)Troponin, could not be included in the study data because they were not available in the files of all patients. The third limitation is the lack of FFR measurements in obstructive CAD, especially in the evaluation of borderline lesions. If these parameters were compared with ischemic and non-ischemic lesions in obstructive CAD patients instead of the SYNTAX score, the results would be much more valuable and perhaps meaningful. Another limitation was that all the factors affecting the relationship between monocytes and HDL HDL-C were not evaluated. Finally, the subtypes of monocytes and HDL HDL-C were not examined, and each of these may have different effects and interactions.

## 5. Conclusions

To the best of our knowledge, this is the first study to have evaluated the combination of pEET with the SII, PAI, and MHR. Although previous studies have emphasized that the SII, PAI, and MHR values relate to CAD and its severity, the results of the current study only showed that elevated MHR was associated with obstructive CAD in patients with pEET and suspected CAD. It has been shown in this study that the combination of the two tests (EET and MHR), which are easily accessible, low-cost, and provide a rapid result, can be used to support the clinician’s suspicion, even if they are not useful for the diagnosis of obstructive CAD. Therefore, elevated MHR in those with pEET can be used as a biomarker before coronary angiography.

## Figures and Tables

**Table 1:** Patient characteristics and laboratory findings.

	Group 1 (n = 434) (NCA and n-obsCAD)	Group 2 (n = 106) (obsCAD)	*p*
Age	54.4 ± 9.6	59.4 ± 9.4	**<0.001**
Sex (F/M)	190/244 (%43.8/%56.2)	21/85 (%19.8/%80.2)	**<0.001**
BMI	30.1 ± 5.2	29.1 ± 4.4	0.067
SBP	117.6 ± 13.6	120.1 ± 15.1	0.128
DBP	76.5 ± 7.8	77.4 ± 7.3	0.292
Heart rate.	86.4 ± 15	87.3 ± 13.8	0.562
HT	88 (%20.3)	16 (%15.1)	0.282
DM	60 (%13.8)	27 (%25.5)	**0.003**
Glucose	102.5 [93–122]	109.5 [95.5–146.8]	**0.006**
GFR	95.3 ± 14.5	90.1 ± 13.5	**0.001**
LDL-C	117.7 ± 35.8	125.9 ± 34.6	**0.033**
Triglyceride	157 [112.8–223]	163 [126.8–249.5]	0.160
HDL-C	43 [37–51]	40.5 [34–47]	**0.010**
WBC	7254.8 ± 1823.8	7666.04 ± 2047.0	**0.043**
Neutrophil	4304.2 ± 1421.3	4541.23 ± 1542.2	0.131
Lymphocyte	2210.1 ± 627.9	2305.3 ± 748.7	0.179
Monocyte	545.1 ± 163.6	577.0 ± 193.5	0.120
Hemoglobin	14.6 ± 1.7	14.9 ± 1.7	0.162
Platelet	255.6 ± 55.5	261.3 ± 70.9	0.446
MHR	13.1 ± 5.5	14.7 ± 6.3	**0.019**
SII	479 [361.8–632.3]	478 [345.3–660.8]	0.848
PAI	0.21 ± 0.269	0.259 ± 0.274	0.094

BMI: body mass index, DBP: diastolic blood pressure, DM: diabetes mellitus, F: female, GFR: glomerular filtration rate, HDL-C: high-density lipoprotein, HT: hypertension, LDL-C: low-density lipoprotein, M: male, MHR: monocyte HDL-C ratio, NCA: normal coronary arteries, n-obsCAD: non-obstructive coronary artery disease, obsCAD: obstructive coronary artery disease, PAI: plasma atherogenic index, SBP: systolic blood pressure, SII: systemic inflammatory index, WBC: white blood cell. *p* values < 0.05 are emphasized in bold.

**Table 2:** Multiple logistic regression analysis results for Table 1.

Factor	Odds Ratio (%95 CI)	*p*
Age	1058 (1034–1084)	**<0.001**
Sex (Male vs. Female)	3652 (2137–6239)	**<0.001**
DM	2239 (1285–3903)	**0.004**
LDL-C	1009 (1002–1015)	**0.007**

DM: diabetes mellitus, LDL-C: low-density lipoprotein, F: female, M: male. *p* values < 0.05 are emphasized in bold.

**Table 3:** Comparison of PAI, SII, and MHR values according to SYNTAX score in patients with obsCAD.

obsCAD	SYNTAX ≤ 22 (n = 82)	SYNTAX 23≤ (n = 24)	*p*
PAI	0.251 ± 0.275	0.290 ± 0.273	0.543
SII	478 [369–652]	471 [293.5–815.3]	0.711
MHR	14.5 ± 6.1	15.3 ± 7.1	0.616

CAD: coronary artery disease, MHR: monocyte HDL-C ratio, obsCAD: obstructive coronary artery disease, PAI: plasma atherogenic index, SII: systemic inflammatory index.

**Table 4:** Coronary angiographic characteristics of patients in the obsCAD group.

(**a**)							
**Treatment Strategy** **n = 106 (%100)**		**OMT** **n = 14 (%13)**		**PCI** **n = 57 (%54)**		**CABG** **n = 35 (%33)**	
(**b**)							
	**Obstructed Vessel n = 189 (%100)**	**LAD** **n = 70 (%37)**	**CX** **n = 59 (%31)**		**RCA** **n = 49 (%26)**	**IMA** **n = 9 (%5)**	**LMCA** **n = 2 (%1)**
**Treatment** **Strategy**							
OMT		8	6		5	2	0
PCI		29	26		24	2	0
CABG		33	27		20	5	2

CABG: coronary artery bypass graft, CX: circumflex artery, IMA: intermediate artery, LAD: left anterior descending artery, LMCA: left main coronary artery, obsCAD: obstructive coronary artery disease, OMT: optimal medical therapy, PCI: percutaneous coronary intervention, RCA: right coronary artery.

## Data Availability

All data generated or analyzed during this study are included in this published article.

## References

[1] Authors/Task Force Members, ESC Committee for Practice Guidelines (CPG), ESC National Cardiac Societies (2019). 2019 ESC/EAS guidelines for the management of dyslipidaemias: Lipid modification to reduce cardiovascular risk. Atherosclerosis.

[2] Knuuti J., Wijns W., Saraste A., Capodanno D., Barbato E., Funck-Brentano C., Prescott E., Storey R.F., Deaton C., Cuisset T. (2020). 2019 ESC Guidelines for the diagnosis and management of chronic coronary syndromes: The Task Force for the diagnosis and management of chronic coronary syndromes of the European Society of Cardiology (ESC). Eur. Heart J..

[3] Gianrossi R., Detrano R., Mulvihill D., Lehmann K., Dubach P., Colombo A., McArthur D., Froelicher V. (1989). Exercise-induced ST depression in the diagnosis of coronary artery disease. A meta-analysis. Circulation.

[4] Roifman I., Wijeysundera H.C., Austin P.C., Rezai M.R., Wright G.A., Tu J.V. (2017). Comparison of Anatomic and Clinical Outcomes in Patients Undergoing Alternative Initial Noninvasive Testing Strategies for the Diagnosis of Stable Coronary Artery Disease. J. Am. Heart Assoc..

[5] Hu B., Yang X.R., Xu Y., Sun Y.F., Sun C., Guo W., Zhang X., Wang W.M., Qiu S.J., Zhou J. (2014). Systemic immune-inflammation index predicts prognosis of patients after curative resection for hepatocellular carcinoma. Clin. Cancer Res..

[6] Yang Y.L., Wu C.H., Hsu P.F., Chen S.C., Huang S.S., Chan W.L., Lin S.J., Chou C.Y., Chen J.W., Pan J.P. (2020). Systemic immune-inflammation index (SII) predicted clinical outcome in patients with coronary artery disease. Eur. J. Clin. Investig..

[7] Gur D.O., Efe M.M., Alpsoy S., Akyüz A., Uslu N., Çelikkol A., Gur O. (2022). Systemic Immune-Inflammatory Index as a Determinant of Atherosclerotic Burden and High-Risk Patients with Acute Coronary Syndromes. Arq. Bras. Cardiol..

[8] Liu Y., Ye T., Chen L., Jin T., Sheng Y., Wu G., Zong G. (2021). Systemic immune-inflammation index predicts the severity of coronary stenosis in patients with coronary heart disease. Coron. Artery Dis..

[9] Dziedzic E.A., Gąsior J.S., Tuzimek A., Paleczny J., Junka A., Dąbrowski M., Jankowski P. (2022). Investigation of the Associations of Novel Inflammatory Biomarkers-Systemic Inflammatory Index (SII) and Systemic Inflammatory Response Index (SIRI)-With the Severity of Coronary Artery Disease and Acute Coronary Syndrome Occurrence. Int. J. Mol. Sci..

[10] Han S.H., Nicholls S.J., Sakuma I., Zhao D., Koh K.K. (2016). Hypertriglyceridemia and Cardiovascular Diseases: Revisited. Korean Circ. J..

[11] Goldbourt U., Yaari S., Medalie J.H. (1997). Isolated low HDL cholesterol as a risk factor for coronary heart disease mortality. A 21-year follow-up of 8000 men. Arterioscler. Thromb. Vasc. Biol..

[12] Shen S., Lu Y., Qi H., Li F., Shen Z., Wu L., Yang C., Wang L., Shui K., Wang Y. (2016). Association between ideal cardiovascular health and the atherogenic index of plasma. Medicine.

[13] Niroumand S., Khajedaluee M., Khadem-Rezaiyan M., Abrishami M., Juya M., Khodaee G., Dadgarmoghaddam M. (2015). Atherogenic Index of Plasma (AIP): A marker of cardiovascular disease. Med. J. Islam Repub. Iran..

[14] Fernández-Macías J.C., Ochoa-Martínez A.C., Varela-Silva J.A., Pérez-Maldonado I.N. (2019). Atherogenic Index of Plasma: Novel Predictive Biomarker for Cardiovascular Illnesses. Arch. Med. Res..

[15] Wu J., Zhou Q., Wei Z., Wei J., Cui M. (2021). Atherogenic Index of Plasma and Coronary Artery Disease in the Adult Population: A Meta-Analysis. Front. Cardiovasc. Med..

[16] Ulloque-Badaracco J.R., Hernandez-Bustamante E.A., Alarcon-Braga E.A., Mosquera-Rojas M.D., Campos-Aspajo A., Salazar-Valdivia F.E., Valdez-Cornejo V.A., Benites-Zapata V.A., Herrera-Añazco P., Valenzuela-Rodríguez G. (2022). Atherogenic index of plasma and coronary artery disease: A systematic review. Open Med..

[17] Jiang M., Yang J., Zou H., Li M., Sun W., Kong X. (2022). Monocyte-to-high-density lipoprotein-cholesterol ratio (MHR) and the risk of all-cause and cardiovascular mortality: A nationwide cohort study in the United States. Lipids Health Dis..

[18] Ganjali S., Gotto A.M., Ruscica M., Atkin S.L., Butler A.E., Banach M., Sahebkar A. (2018). Monocyte-to-HDL-cholesterol ratio as a prognostic marker in cardiovascular diseases. Cell Physiol..

[19] Akboga M.K., Balci K.G., Maden O., Ertem A.G., Kirbas O., Yayla C., Acar B., Aras D., Kisacik H., Aydogdu S. (2016). Usefulness of monocyte to HDL-cholesterol ratio to predict high SYNTAX score in patients with stable coronary artery disease. Biomark Med..

[20] Cetin M.S., Ozcan Cetin E.H., Kalender E., Aydin S., Topaloglu S., Kisacik H.L., Temizhan A. (2016). Monocyte to HDL Cholesterol Ratio Predicts Coronary Artery Disease Severity and Future Major Cardiovascular Adverse Events in Acute Coronary Syndrome. Heart Lung Circ..

[21] Liu H.T., Jiang Z.H., Yang Z.B., Quan X.Q. (2022). Monocyte to high-density lipoprotein ratio predict long-term clinical outcomes in patients with coronary heart disease: A meta-analysis of 9 studies. Medicine.

[22] Kanbay M., Solak Y., Unal H.U., Kurt Y.G., Gok M., Cetinkaya H., Karaman M., Oguz Y., Eyileten T., Vural A. (2014). Monocyte count/HDL cholesterol ratio and cardiovascular events in patients with chronic kidney disease. Int. Urol. Nephrol..

[23] Fletcher G.F., Ades P.A., Kligfield P., Arena R., Balady G.J., Bittner V.A., Coke L.A., Fleg J.L., Forman D.E., Gerber T.C. (2013). Exercise standards for testing and training: A scientific statement from the American Heart Association. Circulation.

[24] Hansson G.K. (2005). Inflammation, atherosclerosis, and coronary artery disease. N. Engl. J. Med..

[25] Tani S., Matsumoto M., Anazawa T., Kawamata H., Furuya S., Takahashi H., Iida K., Washio T., Kumabe N., Kobori M. (2012). Development of a model for prediction of coronary atherosclerotic regression: Evaluation of high-density lipoprotein cholesterol level and peripheral blood monocyte count. Heart Vessels.

[26] Yuan Y., Li P., Ye J. (2012). Lipid homeostasis and the formation of macrophage-derived foam cells in atherosclerosis. Protein Cell.

[27] Ghattas A., Griffiths H.R., Devitt A., Lip G.Y., Shantsila E. (2013). Monocytes in coronary artery disease and atherosclerosis: Where are we now?. J. Am. Coll. Cardiol..

[28] Gratchev A., Sobenin I., Orekhov A., Kzhyshkowska J. (2012). Monocytes as a diagnostic marker of cardiovascular diseases. Immunobiology.

[29] Toth P.P., Barter P.J., Rosenson R.S., Boden W.E., Chapman M.J., Cuchel M., D’Agostino R.B., Davidson M.H., Davidson W.S., Heinecke J.W. (2013). High-density lipoproteins: A consensus statement from the National Lipid Association. J. Clin. Lipidol..

[30] Tardif J.C., Grégoire J., L’Allier P.L., Ibrahim R., Lespérance J., Heinonen T.M., Kouz S., Berry C., Basser R., Lavoie M.A. (2007). Effects of reconstituted high-density lipoprotein infusions on coronary atherosclerosis: A randomized controlled trial. JAMA.

[31] Di Angelantonio E., Sarwar N., Perry P., Kaptoge S., Ray K.K., Thompson A., Wood A.M., Lewington S., Sattar N., Emerging Risk Factors Collaboration (2009). Major lipids, apolipoproteins, and risk of vascular disease. JAMA.

[32] Murphy A.J., Chin-Dusting J.P., Sviridov D., Woollard K.J. (2009). The anti inflammatory effects of high density lipoproteins. Curr. Med. Chem..

[33] Murphy A.J., Woollard K.J. (2010). High-density lipoprotein: A potent inhibitor of inflammation. Clin. Exp. Pharmacol. Physiol..

[34] Zhou Y., Wang L., Jia L., Lu B., Gu G., Bai L., Cui W. (2021). The Monocyte to High-Density Lipoprotein Cholesterol Ratio in the Prediction for Atherosclerosis: A Retrospective Study in Adult Chinese Participants. Lipids.

[35] Yan S., Sha S., Wang D., Li S., Jia Y. (2023). Association between monocyte to high-density lipoprotein ratio and coronary heart disease in US adults in the National Health and Nutrition Examination Surveys 2009–2018. Coron. Artery Dis..

[36] Kundi H., Kiziltunc E., Cetin M., Cicekcioglu H., Cetin Z.G., Cicek G., Ornek E. (2016). Association of monocyte/HDL-C ratio with SYNTAX scores in patients with stable coronary artery disease. Zusammenhang des Monozyten-/HDL-C-Quotienten mit dem SYNTAX-Score bei Patienten mit stabiler koronarer Herzkrankheit. Herz.

[37] Candemir M., Kiziltunç E., Nurkoç S., Şahinarslan A. (2021). Relationship Between Systemic Immune-Inflammation Index (SII) and the Severity of Stable Coronary Artery Disease. Angiology.

[38] Erdoğan M., Erdöl M.A., Öztürk S., Durmaz T. (2020). Systemic immune-inflammation index is a novel marker to predict functionally significant coronary artery stenosis. Biomark Med..

[39] Wang L., Chen F., Xiaoqi C., Yujun C., Zijie L. (2021). Atherogenic Index of Plasma Is an Independent Risk Factor for Coronary Artery Disease and a Higher SYNTAX Score. Angiology.

[40] Newman R.J., Darrow M., Cummings D.M., King V., Whetstone L., Kelly S., Jalonen E. (2008). Predictive value of exercise stress testing in a family medicine population. J. Am. Board Fam. Med..

[41] Taylor C.A., Fonte T.A., Min J.K. (2013). Computational fluid dynamics applied to cardiac computed tomography for noninvasive quantification of fractional flow reserve: Scientific basis. J. Am. Coll. Cardiol..

[42] Budoff M.J., Dowe D., Jollis J.G., Gitter M., Sutherland J., Halamert E., Scherer M., Bellinger R., Martin A., Benton R. (2008). Diagnostic performance of 64-multidetector row coronary computed tomographic angiography for evaluation of coronary artery stenosis in individuals without known coronary artery disease: Results from the prospective multicenter ACCURACY (Assessment by Coronary Computed Tomographic Angiography of Individuals Undergoing Invasive Coronary Angiography) trial. J. Am. Coll. Cardiol..

[43] Li Y., Qiu H., Hou Z., Zheng J., Li J., Yin Y., Gao R. (2022). Additional value of deep learning computed tomographic angiography-based fractional flow reserve in detecting coronary stenosis and predicting outcomes. Acta Radiol..

[44] Greenwood J.P., Maredia N., Younger J.F., Brown J.M., Nixon J., Everett C.C., Bijsterveld P., Ridgway J.P., Radjenovic A., Dickinson C.J. (2012). Cardiovascular magnetic resonance and single-photon emission computed tomography for diagnosis of coronary heart disease (CE-MARC): A prospective trial. Lancet.

[45] Fathala A., Aboulkheir M., Shoukri M.M., Alsergani H. (2019). Diagnostic accuracy of ^13^N-ammonia myocardial perfusion imaging with PET-CT in the detection of coronary artery disease. Cardiovasc. Diagn. Ther..

[46] Woodward W., Dockerill C., McCourt A., Upton R., O’Driscoll J., Balkhausen K., Chandrasekaran B., Firoozan S., Kardos A., Wong K. (2022). Real-world performance and accuracy of stress echocardiography: The EVAREST observational multi-centre study. Eur. Heart J. Cardiovasc. Imaging.

[47] Molinaro A.M. (2015). Diagnostic tests: How to estimate the positive predictive value. Neurooncol. Pract..

[48] Froelicher V.F., Maron D. (1981). Exercise testing and ancillary techniques to screen for coronary heart disease. Prog. Cardiovasc. Dis..

[49] van de Sande D.A., Breuer M.A., Kemps H.M. (2016). Utility of Exercise Electrocardiography in Pre-participation Screening in Asymptomatic Athletes: A Systematic Review. Sports Med..

[50] Levisman J.M., Aspry K., Amsterdam E.A. (2012). Improving the positive predictive value of exercise testing in women for coronary artery disease. Am. J. Cardiol..

